# Doxycycline and Tigecycline: Two Friendly Drugs with a Low Association with *Clostridium Difficile* Infection

**DOI:** 10.3390/antibiotics4020216

**Published:** 2015-06-19

**Authors:** Yuan-Pin Hung, Jen-Chieh Lee, Hsiao-Ju Lin, Hsiao-Chieh Liu, Yi-Hui Wu, Pei-Jane Tsai, Wen-Chien Ko

**Affiliations:** 1 Department of Internal Medicine, Tainan Hospital, Ministry of Health and Welfare, Tainan 70043, Taiwan; E-Mails: yuebin16@yahoo.com.tw (Y.-P.H.); angeltearsjuju@yahoo.com.tw (H.-J.L.); annie800203@yahoo.com.tw (H.-C.L.); 2 Department of Internal Medicine, National Cheng Kung University Hospital, No. 138, Sheng Li Road, Tainan 70403, Taiwan; E-Mail: jclee.eric@msa.hinet.net; 3 Graduate Institute of Clinical Medicine, National Health Research Institutes, Tainan 70403, Taiwan; 4 Department of Medical Laboratory Science and Biotechnology, National Cheng Kung University, Medical College, Tainan 70102, Taiwan; E-Mail: peijtsai@mail.ncku.edu.com; 5 Department of Internal Medicine, E-da Hospital, Kaohsiung 82445, Taiwan; E-Mail: wuyihui@gmail.com; 6 Center of Infectious Disease and Signaling Research, National Cheng Kung University, Tainan 70102, Taiwan; 7 Department of Medicine, National Cheng Kung University, Medical College, Tainan 70102, Taiwan

**Keywords:** *Clostridium difficile*, doxycycline, tigecycline

## Abstract

*Clostridium difficile* infection (CDI) is known to be associated with prior exposure to many classes of antibiotics. Standard therapy for CDI (*i.e.*, metronidazole and vancomycin) is associated with high recurrence rates. Although tetracycline derivatives such as tetracycline, doxycycline or tigecycline are not the standard therapeutic choices for CDI, they may serve as an alternative or a component of combination therapy. Previous tetracycline or doxycycline usage had been shown to have less association with CDI development. Tigecycline, a broad-spectrum glycylcycline with potency against many gram-positive or gram-negative pathogens, had been successfully used to treat severe or refractory CDI. The *in vitro* susceptibility of *C. difficile* clinical isolates to tigecycline in many studies showed low minimal inhibitory concentrations. Tigecycline can suppress *in vitro* toxin production in both historical and hypervirulent *C. difficile* strains and reduce spore production in a dose-dependent manner. Tetracycline compounds such as doxycycline, minocycline, and tigecycline possess anti-inflammatory properties that are independent of their antibiotic activity and may contribute to their therapeutic effect for CDI. Although clinical data are limited, doxycycline is less likely to induce CDI, and tigecycline can be considered one of the therapeutic choices for severe or refractory CDI.

## 1. Clinical Impact of *Clostridium Difficile* Infections

Nosocomial diarrhea is a common complication in hospitalized patients and contributes to increased morbidity and mortality and prolonged hospitalization [1,2]. Infectious diarrhea accounts for 10%–30% of nosocomial diarrhea [3]. *Clostridium difficile* infection (CDI) is the most well-known disease within the etiology of nosocomial infectious diarrhea. Moreover, *C. difficile* is the major cause of nosocomial antibiotic-associated diarrhea through the production of toxins A and B. The CDI disease pattern ranges from mild diarrhea to pseudomembranous colitis, toxic megacolon or colon perforation [4]. The 30-day attributable mortality rate in CDI cases is 6.9%; this rate is higher in patients who developed toxic megacolon, ranging from 25% to 40% [5,6]. *C. difficile* is frequently transmitted in healthcare settings via medical care workers. Therefore, CDI represents an important infection control issue [7]. 

The incidence of CDI has been increasing in recent years with sporadic outbreaks. The most well-known event was the CDI outbreak in Quebec, Canada in 2003. In this outbreak, CDI was the attributable cause of death in 117 (6.9%) cases out of 1703 patients and was a contributing factor in an additional 127 (7.5%) deaths [6]. In the United States, the number of CDI cases reported in 2005 (84 per 100,000) was nearly three times the number reported in 1996 (31 per 100,000) [4]. A recent multistate active surveillance study in the US revealed alarming national data concerning CDI that was indicative of a persistent health threat. In 2011, the estimated number of incident CDI cases was more than 453,000, and the crude incidence of community-associated and healthcare-associated CDI cases was 48.2 and 92.8 episodes per 100,000 persons in the US, respectively [8]. Of greater concern is the increase in severe and fatal infections [4]. The incidence of CDI at a medical center in southern Taiwan was 42.6 cases per 100,000 patient-days or 3.4 cases per 1000 discharges between January 2007 and March 2008 [9]. Notably, the incidence of toxigenic *C. difficile* colonization was higher, accounting for 84.8 cases/100,000 patient-days or 73.0/1000 patients at a regional hospital from 2011 to 2012 [10].

## 2. Antibiotic Exposure Related to the Development of *Clostridium Difficile* Infection

Antibiotic exposure causes disruption of the normal colonic flora and predisposes patients to *C. difficile* colonization and infection [9,10,11,12]. Antibiotics decrease the density of intestinal bacteria and alter the intestinal microbiota. The effect may last for more than eight weeks after the cessation of antibiotics [13]. Exposure to more than one class of antibiotics resulted in a higher rate of *C. difficile* colonization [14]. Notorious antimicrobial agents that have been linked to the disruption of the indigenous microflora [15] and CDI development include third-generation cephalosporins [16,17], clindamycin [16,17,18], and fluoroquinolones [17,18,19,20]. These drugs can be regarded as “predisposing” drugs. Current clinical management for CDI involves the discontinuation of predisposing antibiotics and the administration of anti-CDI antibiotics, such as metronidazole and vancomycin [4].

Interestingly, some antibiotics, such as piperacillin-tazobactam, exhibited *in vitro* antibacterial activity against clinical *C. difficile* strains. Moreover, prior exposure to these antibiotics was noted to be less likely to induce *Clostridium difficile*-associated diarrhea (CDAD). These antibiotics were once considered to be “friendly” antibiotics [21]. For example, a higher incidence of CDI was noted in patients with *C. difficile* colonization who received cefotaxime as opposed to piperacillin-tazobactam (69.2%, 18/26 *versus* 33.3%, 1/3) [22]. Furthermore, the rate of CDI increased following the shortage of piperacillin-tazobactam [23]. Nevertheless, the classification of piperacillin-tazobactam as a “friendly” antibiotic was challenged because opposing results were reported in other studies [17,24,25]. Although piperacillin-tazobactam can inhibit *C. difficile in vitro*, it most likely disrupts the anaerobic gut microflora and facilitates the growth of *C. difficile* [24]. Indeed, the administration of penicillins and β-lactamase inhibitor combinations (mainly piperacillin-tazobactam) has been recognized to be associated with an increased risk of CDI [17]. A significant reduction in the rate of CDI was noted with the reduced availability of piperacillin-tazobactam [25]. Such contradictory information indicates that the interaction of antibiotics, the gut microbiota, and pathogenic *C. difficile* is complex in the clinical real world.

Even metronidazole, a therapeutic drug for CDI that had been considered for prophylaxis for CDI in high risk patients [26], might not be free of risk because its use has been associated with CDI relapse (odds ratio, 2.74) [27]. Another finding arguing against the preventive potential of metronidazole is the occurrence of CDI after the administration of a triple metronidazole-containing regimen to eradicate *Helicobacter pylori* [28]. Thus, exposure to broad-spectrum antibiotics that are active against *C. difficile in vitro* may predispose a susceptible patient to CDI, most likely due to inadequate anti-clostridial activity and the loss of colonization resistance in the gut.

## 3. Doxycycline—Varied Susceptibility Data *In Vitro* but High Gut Tissue Concentrations

Before the susceptibility data of tetracycline for *C. difficile* isolates can be interpreted and compared, the important issue of the susceptible or resistant breakpoint must be addressed. No susceptible or resistant breakpoint for doxycycline was recommended from the Clinical and Laboratory Standards Institutes (CLSI), British Society for Antimicrobial Chemotherapy (BSAC) or European Committee on Antimicrobial Susceptibility Testing (EUCAST). Moreover, the susceptibility of tetracycline or its derivatives has rarely been reported for *C. difficile* isolates, making international comparisons difficult. This lack is at least partially related to the limited pharmacological data available for doxycycline at the target site (the intestinal mucosa) and the lack of a correlation between clinical treatment and the therapeutic outcome of doxycycline, which is not commonly used for CDI.

Although the susceptibility of *C. difficile* isolates to tetracycline varied geographically, most isolates reported in Europe were susceptible to tetracycline derivatives, with resistance rates of less than 10% [29,30,31,32]. The resistance rates of doxycycline in *C. difficile* isolates from animal products, animal stools, or soil were low, ranging from 0% to 2.1% as shown in Table 1 [33,34,35]. However, the susceptibility data for clinical *C. difficile* isolates varied significantly in a limited number of reports. A total of 11% of 43 isolates from Spain were doxycycline-resistant using a resistance criterion of minimal inhibitory concentration (MIC) >2 μg/mL [36]. Doxycycline resistance defined as a doxycycline MIC ≥8 μg/mL was noted in nearly 40% of 317 clinical isolates from Germany [37]. In contrast, all 48 clinical isolates in Israel were susceptible to doxycycline (MIC range: 0.016–0.38 g/mL) [38]. Even with the existence of a substantial degree of doxycycline resistance in clinical strains, a preventive or therapeutic role for doxycycline in *C. difficile* infection cannot be excluded completely because high doxycycline concentrations are present in bowel tissue after oral administration. Oral intake of 200 mg of doxycycline every 4–6 h resulted in a serum concentration of 4.0 ± 0.3 mg/L, ileum concentration of 7.5 ± 1.2 mg/L, and colon concentration of 3.9 ± 0.3 mg/L [39]. Theoretically, the high bowel tissue concentration may render doxycycline effective against *C. difficile* strains with higher MICs, but the drug levels in the intestinal lumen where *C. difficile* vegetative cells reside is likely to be the critical variable for the prevention or treatment of CDI.

## 4. Doxycycline—Protective Effect against CDI in Clinics?

Some reports have discussed the connection between doxycycline and CDI [40,41,42], but no information is available concerning minocycline. Although CDI was noted after doxycycline prophylaxis for malaria [43], doxycycline was associated with a decreased incidence of CDI in recent studies. Doxycycline was associated with a reduced risk of CDI (odds ratio, 0.41) in a retrospective case-control study that compared 1142 cases of hospital-acquired CDI from 1999 through 2005 with 3351 controls matched for facility [40]. In another study that used a multivariable model to adjust for confounding factors, the rate of CDI was 27% lower for each day of doxycycline administration compared to a patient without doxycycline therapy (hazard ratio, 0.73) [41]. A pharmacotherapy review concluded that doxycycline had protective effects against the development of CDI [42]. In contrast to the studies in Europe, tetracycline resistance was present in more than 30% of *C. difficile* isolates in China [32,44,45,46]. Thus, the preventive effect of tetracycline derivatives for CDI warrants confirmation by further clinical observation.

**Table 1:** *In vitro* susceptibility of doxycycline and tigecycline against *Clostridium difficile* isolates.

Authors	Year	Location	Isolate Numbers ^3^	Test Method	MIC, μg/mL			Resistant Rate, % (Breakpoint)	Reference
					MIC_50_	MIC_90_	Range		
Doxycycline									
Schmidt *et al.*	2002–2004	Germany	317	Etest	0.125	32	<0.06–>256	37 (≥8 μg/mL)	[35]
Bishara *et al.*	2003–2004 ^2^	Israel	49	Disc diffusion and Etest	0.016	0.023	0.016–0.38	0 (ND)	[36]
Simango *et al.*	2006 ^2^	Zimbabwe	53 from soil and animal feces	Disc diffusion	ND	ND	ND	0 (ND)	[33]
Simango *et al.*	2008 ^2^	Zimbabwe	51 from chicken	Disc diffusion	ND	ND	ND	0 (ND)	[32]
Kouassi *et al.*	2009–2010	Cote d’Ivoire	49 from beef	Disc diffusion	ND	ND	ND	2.1 (<17 mm)	[31]
Alcalá *et al.*	2012^2^	Spain	43	Etest	0.032	3	0.016–8	11.4 (≥16 μg/mL)	[34]
**Tigecycline** ^1^									
Hecht *et al.*	1983–2004	Primarily from the US	110	Agar dilution	0.125	0.25	0.06–1	ND	[46]
Edlund *et al.*	1998	Sweden	50	Agar dilution	0.032	0.032	0.016–0.032	ND	[47]
Hawser *et al.*	2008	Europe	256	Agar dilution	<0.06	0.25	0.06–2	5.1	[48]
Rashid *et al.*	2008–2011	Stockholm, Sweden	114	Agar dilution	0.064	0.125	0.032–0.25	0	[27]
Lin *et al.*	2011	Taiwan	108	Agar dilution	0.06	0.06	0.03–0.25	0	[45]
Lachowicz *et al.*	2012	Poland	83	Etest	0.094	0.19	0.016–0.25	0	[49]
Rashid *et al.*	2013^2^	Stockholm, Sweden	133	Agar dilution	0.064	0.125	0.032–0.25	0	[28]

^1^ Resistant breakpoint of the European Committee on Antimicrobial Susceptibility Testing (EUCAST) for tigecycline: >0.5 μg/mL; ^2^ Publication year; ^3^ Clinical isolates, if not specified. ND: no data.

## 5. Tigecycline—Good *In Vitro* Susceptibility but Bad for Gut Microbiota

Tigecycline is a new generation tetracycline derivative. Although there is no CLSI breakpoint of tigecycline for *C. difficile*, *in vitro* susceptibility studies of clinical *C. difficile* isolates to tigecycline showed excellent antibacterial activity [29,30,47,48,49,50,51] (Table 1). For example, two (2.4%) strains from a collection of 83 toxigenic *C. difficile* isolates from Polish hospitals were resistant to tetracycline, while all of the strains were sensitive to tigecycline according to the latest EUCAST breakpoint (MIC <0.25 μg/mL).

Although tigecycline has the capability to inhibit toxin production or spore formation, its broad-spectrum antibacterial activity on gut microbiota renders its usage a risk factor for CDI. Tigecycline treatment resulted in major shifts in the gut microbiota in mice, including a decrease in *Bacteroidetes* levels and an increase in *Proteobacteria* levels, which made the treated mice susceptible to CDI [52]. Such a change in gut microbiota could last for weeks, as evidenced by the findings that the recovery of the bacterial community was incomplete and diversity was lower compared to untreated controls 5 weeks after the cessation of tigecycline treatment [52]. However, tigecycline instillation reduced competing microflora, including *Bacteroides* and *Bifidobacteria*, in an *in vitro* human gut model but did not induce *C. difficile* proliferation or cytotoxin production [53].

## 6. Tigecycline—Beyond Antibacterial Effects against *C. Difficile*

Notably, tigecycline can suppress spore formation *in vitro* [24], and this finding was linked to the potential efficacy for tigecycline therapy against recurrent CDI. Furthermore, tigecycline did not promote the growth or toxin production of *C. difficile* in a mouse model, and concurrent administration of tigecycline prevented clindamycin-induced promotion of *C. difficile* growth in the cecal contents [54]. Tigecycline can suppress *in vitro* toxin production in both historical and hypervirulent *C. difficile* strains and reduce spore production in a dose-dependent manner [55,56]. These experimental data partially explain the fact that tigecycline therapy was associated with a low recurrence rate of CDI [57,58]. In contrast, metronidazole and vancomycin, two standard antimicrobial drugs for CDI, had a similar suppressive effect on toxin production but less suppression on spore formation [55], which provided an explanation as to why metronidazole or vancomycin therapy for CDI was related to a higher recurrence rate [59,60].

Tetracycline compounds possess anti-inflammatory properties independent of their antibiotic activity that may contribute to their therapeutic effects on CDI [61]. Tigecycline can attenuate staphylococcal superantigen-induced T-cell proliferation and the production of cytokines (*i.e.*, IL-1β, IL-6, and TNF-α) and chemokines (*i.e.*, MIP-1α and MIP-1β) [62]. For example, tigecycline therapy significantly reduced the concentrations of inflammatory pulmonary cytokine (*i.e.*, IL-1β, IL-12, IFN-γ, and TNF-α) and chemokine concentrations (*i.e.*, MIG, MIP-1α, and IP-10) in a murine model of *Mycoplasma pneumoniae* pneumonia [63]. Moreover, tigecycline prevented the lipopolysaccharide-induced release of pro-inflammatory and apoptotic mediators in neuronal cells [64], thereby exhibiting a so-called “neuroprotective effect”. In CDI cases, these anti-inflammatory effects may contribute additional therapeutic benefits to intestinal inflammation.

## 7. Tigecycline—Treatment of CDI

Metronidazole and vancomycin are currently regarded as the primary therapy for CDI, although the choice of initial therapy depends on the severity of disease. Metronidazole is the agent of choice for most patients with mild to moderate CDI and can be administered via the oral or intravenous routes [65]. Oral vancomycin is recommended for patients with severe CDI [65]. The facilitation of fecal colonization by vancomycin-resistant enterococci is a potential drawback of oral vancomycin [66]. Nevertheless, recurrent CDI was noted with either metronidazole or vancomycin therapy and was more difficult to manage than the initial CDI episodes [59,60]. The newer antibiotic fidaxomicin showed promising therapeutic results, with clinical cure rates similar to those of vancomycin and lower recurrence rates [67]. Nevertheless, fidaxomicin is expensive and not available worldwide.

Although tetracycline derivatives such as doxycycline or tigecycline are not the standard therapeutic choices for CDI, they may serve as alternatives or components of combination therapy for refractory CDI. Although tigecycline usage might be a risk factor for CDI, successful treatment of CDI using a tigecycline-containing combination regimen had been reported. The dosage of tigecycline commonly used was an intravenous loading dose of 100 mg, followed by 50 mg every 12 h. In 2009, Herpers and colleagues first described four cases of severe refractory CDI that were successfully treated by tigecycline monotherapy or combination therapy with oral vancomycin [68]. Then, Lu successfully treated another case of severe and refractory CDI with tigecycline and metronidazole for 14 days [69]. One patient with recurrent CDI that was refractory to vancomycin and metronidazole was effectively managed by the combination of intravenous tigecycline, oral rifaximin and oral vancomycin [70], while a second patient was managed by intravenous tigecycline and oral rifaximin [71]. Recently, seven cases of severe CDI reported by Nicholas *et al.* were treated with an antibiotic cocktail containing intravenous tigecycline and metronidazole in combination with oral vancomycin, which led to clinical cure in six (85.7%) cases. The sustained response at 28 days was 100% among five evaluable cases [72] (Table 2). Thus, these clinical experiences indicated that tigecycline could be a potential component of combination therapy for CDI, especially in severe or refractory cases.

**Table 2:** Clinical reports of the therapeutic efficacy of tigecycline for *Clostridium difficile* infection (CDI).

Publication Year	Case No.	Severity of CDI ^1^	Duration of Tigecycline, Therapy, Days	Combination Antibiotics	Outcomes	Favor Tigecycline Therapy	Reference
2009	4	Severe	7–24 ^2^	Monotherapy or with oral vancomycin	Clinical improvement	Yes	[59]
2010	1	Severe	14	Oral metronidazole	Clinical improvement	Yes	[60]
2010	1	Severe	18	Intravenous metronidazole and vancomycin enema	Lack of clinical improvement	No	[71]
2012	1	Severe/recurrent	10	Oral rifaximin	Clinical improvement	Yes	[68]
2012	1	Severe/recurrent	4	Oral rifaximin and vancomycin	Clinical improvement	Yes	[67]
2014	43	Severe	No data	Intravenous metronidazole and oral vancomycin	No extra-benefit in requiring colectomy, recurrence or mortality	No	[70]
2014	7	Severe/complicated	3–21	Intravenous metronidazole and oral vancomycin	Clinical improvement in 85.7% of 7 cases	Yes	[69]

^1^ Defined as a white blood cell count >15,000/μL or a rise in serum creatinine to 150% of the premorbid level; severe complicated disease defined as the presence of *C. difficile* sepsis, ileus, or toxic megacolon; ^2^ One patient received tigecycline at a standard dosage for 24 days, followed by an additional two weeks of tigecycline treatment interspersed with one treatment-free week.

In contrast, conflicting clinical data had argued against the use of tigecycline-containing regimens for CDI. Ashley *et al.* compared 18 patients with severe CDI treated with tigecycline-containing regimens in which tigecycline was administered for at least 48 h, 17 patients administered oral vancomycin and intravenous metronidazole and one patient administered oral fidaxomicin compared with 26 patients receiving oral vancomycin and metronidazole therapy [73]. There were no differences in the need for colectomy, recurrence, or mortality between the two groups. Kopterides *et al.* reported a 70-year-old man with severe CDI that eventually succumbed to complications of his illness despite three-week tigecycline therapy in conjunction with vancomycin, metronidazole and intravenous immunoglobulin. Additionally, the specific challenges related to tigecycline therapy (*i.e.*, the development of *Proteus mirabilis* bacteremia and colonization with tigecycline-resistant *Acinetobacter baumannii*) should not be ignored [74].

## 8. Conclusions

In conclusion, prior exposure to doxycycline is less likely to induce CDI. The correlation between prior tigecycline therapy and subsequent CDI is controversial. Nevertheless, tigecycline has an inhibitory effect on toxin production, spore formation, and inflammation and may serve as a potential component of combination therapy for severe or refractory CDI.
